# Editorial: The Long Road to Building a Head: Smooth Travels and Accidents on the Journey From Patterning *via* Morphogenesis to Phenotype

**DOI:** 10.3389/fcell.2022.895497

**Published:** 2022-04-25

**Authors:** Kerstin Feistel, Annette Hammes, Dalit Sela-Donenfeld

**Affiliations:** ^1^ Department of Zoology, Institute of Biology, University of Hohenheim, Stuttgart, Germany; ^2^ Max Delbrück Center for Molecular Medicine in the Helmholtz Association, Berlin, Germany; ^3^ Faculty of Agriculture, Food and Environmental Sciences, Koret School of Veterinary Medicine, The Hebrew University of Jerusalem, Rehovot, Israel

**Keywords:** neural plate, neural crest, cranial placodes, head, vertebrates, brain development, craniofacial malformations, congenital disorders

## Introduction

Formation of the vertebrate head is fascinating in its complexity. Originating from ectodermal, mesodermal and endodermal cell lineages, multiple cell types will initially become specified to neural ectoderm, neural crest cells, sensory placode, head mesoderm and the upper-most part of the gastrointestinal tract (Santagati and Rijli, 2003; Thawani and Groves, 2020). These cell types further differentiate to eventually generate the brain, skull bones and cartilage, sensory organs, facial muscles and connective tissues as well as the pharynx. The development of all these structures has to be tightly orchestrated in order to assemble a functional head, which is critical for survival and communication in all vertebrates (Gans and Northcutt, 1983).

Activation of multiple biochemical and mechanical cues in the early embryonic head initiates a crosstalk between different signaling pathways that elicits cell-autonomous and non-autonomous mechanisms to activate complex gene regulatory networks. By regulating the activity of intermediate players, extensive patterning and specification events eventually trigger morphogenetic cell movements such as apical constriction, cell elongation, cell clustering, epithelial-to-mesenchymal transition and cell migration (Gilmour et al., 2017). It is the intricate temporal and spatial orchestration of these morphogenetic dynamics that brings about neural tube closure, creates additional layers from the pseudostratified cranial neuroectoderm to form different brain regions, facilitates neural crest delamination and migration, promotes accumulation of ectodermal cells to form sensory placodes, as well as enables the mesodermal and endodermal cell layer to shape muscles, connective tissues, blood vessels and endodermal lining. Accidents happening anywhere and anytime on that developmental road can impair head morphogenesis, leading to numerous types of craniofacial malformations and neurodevelopmental disorders (LaMantia, 2020).

## Complex Congenital Malformations of Head Development

In humans, such accidents during embryonic or fetal development often manifest as complex congenital malformations affecting head and brain development. Autosomal recessive primary microcephaly (MCPH) is a common congenital disorder leading to brain atrophy. Zaqout and Kaindl review the rapidly increasing knowledge on genes associated with MCPH and highlight that the expanding pathomechanism spectrum of this disease goes far beyond the well-established centrosomal component (Zaqout and Kaindl). The authors point out the need for deeper understanding of the MCPH genotype-to-phenotype correlation to elucidate molecular pathways involved in disease etiology. Another common congenital malformation of head and brain is holoprosencephaly (HPE), characterized by failure of proper forebrain hemisphere separation and craniofacial dysmorphologies of varying severity. Lo et al. summarize the interactions between genes and environment that underlie the multifactorial etiology of HPE. Focusing on the SHH pathway, their review emphasizes the need to better understand genetic and environmental factors contributing to HPE to shed light on the mechanisms underlying disease etiology.

## Animal Models to Study Brain and Craniofacial Malformations

Animal models are especially fruitful in analyzing the pathomechanisms underlying craniofacial malformations. Severe craniofacial malformations such as extreme microphthalmia and midline facial clefting occur in Frontonasal Dysplasia (FND). Iyyanar et al. provide new insights into the genetic mechanisms underlying the etiology of FND using a mouse model. The authors analyze ALX1-related FND in the mouse and demonstrate requirement of the ALX1 transcription factor for correct patterning of neural crest-derived periocular and frontonasal mesenchyme–cell populations that contribute to cartilage and bones in the skull. Schreiner et al. have used the African Clawed Frog *Xenopus laevis* as a vertebrate embryonic model to analyse the craniofacial aspects of Diamond-Blackfan anemia (DBA). Loss of ribosomal protein L5 (RPL5), which is mutated in patients suffering from DBA, caused malformations of the brain, eyes and cranial cartilage, reflecting the craniofacial defects seen in DBA. *Xenopus* has previously been used to analyze the role of the Autism Susceptibility Candidate 2 (AUTS2) gene and its paralog Fibrosin-like protein 1 (FBRSL1) in the etiology of congenital head and brain pathologies. Here, Pauli et al. review recent studies, discuss the transcriptional complexity of both factors and speculate on how FBRSL1 and AUTS2 might interrelate to govern brain and head development.

## Signaling Pathways in Head Development

The road to head formation can get bumpy as early as during the first patterning events that specify anterior tissues. Analysis of signaling pathways in head development is thus crucial to understand pathomechanisms all the way from patterning to morphogenetic movements. WNT signaling is one of the essential pathways during all stages of head development–from early anterior-posterior patterning of the neural plate *via* neural plate border specification to neural crest migration. While Sutton et al. summarize the functions of WNT signaling in zebrafish neural crest development from specification through neural crest cell migration, Bou-Rouphael and Durand focus on the role of TCF/LEF as transcriptional repressors associated with canonical WNT signaling activity in various contexts relating to head development, from the Spemann organizer to brain organizers, but also in stem cell homeostasis and cancer. In addition to WNT, the BMP and FGF signaling pathways are essential for all stages of head development. Washausen and Knabe highlight their relevance for morphogenesis and neurogenesis in the three paired epibranchial placodes. Using whole mouse embryo culture, the authors show that the development of each epibranchial placode is individually regulated by differential sensitivity to these morphogen pathways. While paracrine signaling by BMP, FGF and WNT establishes tissue-wide patterning, juxtacrine signaling is used for cell segregation in the formation of tissue boundaries. Wilkinson’s review provides an in-depth summary on juxtacrine Eph-Ephrin signaling and its interplay with cadherins for segregation of cells for boundary formation in the developing hindbrain.

## Gene Regulatory Networks in Head Development

Complex gene regulatory networks are initiated by signaling pathways and govern patterning and cell segregation, e.g. at the neural plate border. Klein et al. study ectoderm segregation into neural plate, neural crest, pre-placodal and *epidermis* lineages using *Xenopus*. The authors show that segregation is initially produced by cell autonomous repressive transcription factor interactions, followed by non-cell autonomous signalling to neighbouring cells/domains. The issue of transcriptional regulation in cranial neural crest cell specification and craniofacial morphogenesis was further elaborated by McMahon et al. The authors knocked out the transcription factor Foxd4 in mouse ESCs and used these cells to generate organoids and chimeric mice. Their analysis suggests that within the regulatory network driving head formation, Foxd1 acts at different times in different tissues. First, Foxd4 in the anterior mesendoderm induces anterior neural tissue and subsequently, it becomes essential in anterior neuroectoderm, regulating cranial neural crest development. This article illustrates that the same transcription factor can function in different lineages at consecutive time points during development and contribute to the formation of distinct tissues.

In summary, the goal of this Research Topic was to gather novel data and discussion about the diverse aspects of vertebrate head development in health and disease. By integrating recent advances on the study of congenital disorders affecting head formation with studies on signaling pathways, gene regulatory networks and mechanisms of cell behavior acting in craniofacial development, this Research Topic provides a wide synopsis on the multiple processes that govern head/brain formation during normal development as well as on molecular pathomechanisms that cause complex developmental defects.
